# Supplementary material for: Adherence to the Mediterranean Diet in Women and Reproductive Health across the Lifespan: A Narrative Review
Source: Nutrients. 2023 Apr 28;15(9):2131. doi: 10.3390/nu15092131 (PMC10181412; doi:10.3390/nu15092131)
Supplement: Supplementary file 1 [file nutrients-15-02131-s001.zip › nutrients-2359900-supplementary.docx]

**SUPPLEMENTARY MATERIAL**

**Adherence to the Mediterranean Diet in Women and Reproductive Health across Lifespan: A Narrative Review**

Maria Karolina Szmidt^1*^, Dominka Granda^2^, Dawid Madej^1^, Ewa Sicinska^1^, Joanna Kaluza^1^*

Affiliation:

^1^Department of Human Nutrition, Warsaw University of Life Sciences (WULS-SGGW), Institute of Human Nutrition Sciences, Nowoursynowska 166, 02-787 Warsaw, Poland

^2^Department of Nutrition Physiology and Dietetics, Institute of Sport, National Research Institute, Trylogii 2/16, 01-982 Warsaw, Poland

***Corresponding authors:**

Maria Karolina Szmidt, Institute of Human Nutrition Sciences, Warsaw University of Life Sciences – SGGW, 159C Nowoursynowska Str., 02-776 Warsaw, Poland, E-mail: maria_szmidt@sggw.edu.pl, Phone: +48 22 59 37 119

Joanna Kaluza, Institute of Human Nutrition Sciences, Warsaw University of Life Sciences – SGGW, 159C Nowoursynowska Str., 02-776 Warsaw, Poland, E-mail: joanna_kaluza@sggw.edu.pl, Phone: +48 22 59 37 124

**Supplementary Table 1.** Details of quality assessment of case-control studies with the use of the Newcastle-Ottawa Quality Assessment Scale [8]

| **Authors**  (year) | **Is the case definition adequate^a^** | **Representativeness of the cases** | **Selection of Controls** | **Definition of Controls** | **SELECTION (max. 4 points)** | **Comparability for the most important factor - age** | **Comparability for any additional factor** | **COMPARABILITY (max. 2 points)** | **Ascertainment of exposure** | **Same method of ascertainment for cases and controls** | **Non-response rate** | **EXPOSURE (max. 3 points)** | **TOTAL POINTS (max. 9 points)** |
| --- | --- | --- | --- | --- | --- | --- | --- | --- | --- | --- | --- | --- | --- |
| **Polycystic ovary syndrome** | | | | | | | | | | | | | |
| **Cutilas-Tollin et al.** (2021) | 1 | 1 | 1 | 1 | **4** | 0 | 1 | **1** | 1 | 1 | 0 | **2** | **7** |
| **Wang et al.**  (2022) | 1 | 0 | 1 | 1 | **3** | 0 | 0 | **0** | 1 | 1 | 0 | **2** | **5** |

Low-quality study: 0 to 3 points; Medium-quality study: 4 to 6 points; High-quality study: 7 to 9 points, ^a^medical diagnosis based on the examination before entering to the study

**Supplementary Table 2.** Details of quality assessment of cross-sectional studies with the use of the Newcastle-Ottawa Quality Assessment Scale [8]

| **Authors**  (year) | **Representativeness of the sample** | **Sample size** | **Non-respondents** | **Ascertainment of the exposure** | **SELECTION (max. 5 points)** | **Comparability for the most important factor - age** | **Comparability for any additional factor** | **COMPARABILITY (max. 2 points)** | **Assessment of the outcome** | **Statistical test** | **OUTCOME (max. 3 points)** | **TOTAL POINTS (max.10 points)** |  |
| --- | --- | --- | --- | --- | --- | --- | --- | --- | --- | --- | --- | --- | --- |
| **Premenstrual syndrome** | | | | | | | | | | | | | |
| **Kwon et al.**  (2022) | 1 | 0 | 0 | 2 | **3** | 0 | 0 | **0** | 1 | 1 | **2** | **5** |  |
| **Dysmenorrhea** | | | | | | | | | | | | | |
| **Onieva-Zafra et al.** (2020) | 1 | 0 | 0 | 2 | **3** | 0 | 0 | **0** | 1 | 1 | **2** | **5** |  |
| **Sexual dysfunction** | | | | | | | | | | | | | |
| **Giugliano et al.** (2010) | 0 | 0 | 1 | 1 | **2** | 1 | 1 | **2** | 1 | 1 | **2** | **6** |  |
| **Polycystic ovary syndrome** | | | | | | | | | | | | | |
| **Barrea et al.**  (2019) | 0 | 1 | 0 | 2 | **3** | 1 | 1 | **2** | 1 | 1 | **2** | **7** |  |
| **Barrea et al.**  (2021) | 0 | 1 | 1 | 2 | **4** | 1 | 1 | **2** | 2 | 1 | **3** | **9** |  |
| **Moran et al.**  (2015) | 1 | 0 | 1 | 2 | **4** | 1 | 1 | **2** | 0 | 1 | **1** | **7** |  |
| **Infertility** | | | | | | | | | | | | | |
| **Vujkovic et al.**  (2010) | 0 | 0 | 0 | 2 | **2** | 1 | 1 | **2** | 2 | 1 | **3** | **7** |  |

Low-quality study: 0 to 3 points; Medium-quality study: 4 to 6 points; High-quality study: 7 to 10 points

**Supplementary Table 3.** Details of quality assessment of cohort studies with the use of the Newcastle-Ottawa Quality Assessment Scale [8]

| **Authors**  (year) | | **Representativeness of the exposed cohort** | **Selection of the non-exposed cohort** | **Ascertainment of exposure** | **Demonstration that outcome of interest was not present at start of study** | **SELECTION (max.4 points)** | **Comparability for the most important factor - age** | **Comparability for any additional factor** | **COMPARABILITY (max. 2 points)** | **Assessment of the outcome** | **Was follow-up long enough for outcomes to occur** | **Adequacy of follow-up of cohorts** | **OUTCOME (max. 3 points)** | **TOTAL POINTS (max. 9 points)** |  |  |  |  |  |  |  |  |  |  |  |  |
| --- | --- | --- | --- | --- | --- | --- | --- | --- | --- | --- | --- | --- | --- | --- | --- | --- | --- | --- | --- | --- | --- | --- | --- | --- | --- | --- |
|  | **Menarche** | | | | | | | | | | | | | |  |  |  |  |  |  |  |  |  |  |  |  |
| **Szamreta et al.**  (2019) | | 1 | 1 | 1 | 1 | **4** | 1 | 1 | **2** | 0 | 0 | 1 | 1 | **7** |  |  |  |  |  |  |  |  |  |  |  |  |
|  | **Infertility** | | | | | | | | | | | | | |  |  |  |  |  |  |  |  |  |  |  | **Infertility** |
| **Gaskins et al.** (2014) | | 1 | 1 | 1 | 0 | **3** | 1 | 1 | **2** | 0 | 1 | 1 | 2 | **7** |  |  |  |  |  |  |  |  |  |  |  |  |
| **Gaskins et al.**  (2019) | | 0 | 1 | 0 | 1 | **2** | 1 | 1 | **2** | 1 | 1 | 0 | 2 | **6** |  |  |  |  |  |  |  |  |  |  |  |  |
| **Karayiannis et al.**  (2018) | | 0 | 1 | 1 | 1 | **3** | 1 | 1 | **2** | 1 | 1 | 0 | 2 | **7** |  |  |  |  |  |  |  |  |  |  |  |  |
| **Ricci et al.**  (2019) | | 0 | 1 | 0 | 1 | **2** | 1 | 1 | **2** | 1 | 1 | 1 | 3 | **7** |  |  |  |  |  |  |  |  |  |  |  |  |
| **Sun et al.**  (2019) | | 0 | 1 | 1 | 1 | **3** | 1 | 1 | **2** | 1 | 1 | 1 | 3 | **8** |  |  |  |  |  |  |  |  |  |  |  |  |

Poor quality study(low-quality): 0 or 1 star in selection domain OR 0 stars in comparability domain OR 0 or 1 stars in outcome/exposure domain; Fair quality study (medium-quality): 2 stars in selection domain AND 1 or 2 stars in comparability domain AND 2 or 3 stars in outcome/exposure domain; Good quality stdudy (high-quality): 3 or 4 stars in selection domain AND 1 or 2 stars in comparability domain AND 2 or 3 stars in outcome/exposure domain

**Supplementary Table 4.** Details of quality assessment of RCT with the use of the CASP Randomized Controlled Trial Standard Checklist [9]

|  | **Authors, year** | | | | |
| --- | --- | --- | --- | --- | --- |
|  | **Esposito et al. 2007** | **Maiorino et al. 2016** | **Ott et al.**  **2012** | **Cincione et al. 2022** | **Mei et al.**  **2022** |
| **SECTION A:**  Is the basic study design valid for a randomised controlled trial? | | | | | |
| **Did the study address a clearly focused research question?** | | | | | |
| Yes | **x** |  |  | **x** | **x** |
| No |  | **x** | **x** |  |  |
| Can’t tell |  |  |  |  |  |
| **Was the assignment of participants to interventions randomised?** | | | | | |
| Yes |  | **x** |  | **x** | **x** |
| No |  |  | **x** |  |  |
| Can’t tell | **x** |  |  |  |  |
| **Were all participants who entered the study accounted for at its conclusion?** | | | | | |
| Yes |  | **x** |  |  |  |
| No | **x** |  |  | **x** | **x** |
| Can’t tell |  |  | **x** |  |  |
| **SECTION B:**  Is the basic study design valid for a randomised controlled trial? | | | | | |
| **Were the participants ‘blind’ to intervention they were given?** | | | | | |
| Yes |  | **x** |  |  |  |
| No | **x** |  | **x** | **x** |  |
| Can’t tell |  |  |  |  | **x** |
| **Were the investigators ‘blind’ to the intervention they were giving to participants?** | | | | | |
| Yes |  | **x** |  |  |  |
| No | **x** |  | **x** | **x** |  |
| Can’t tell |  |  |  |  | **x** |
| **Were the people assessing/analysing outcome/s ‘blinded’?** | | | | | |
| Yes |  | **x** |  |  |  |
| No | **x** |  | **x** | **x** |  |
| Can’t tell |  |  |  |  | **x** |
| **Were the study groups similar at the start of the randomised controlled trial?** | | | | | |
| Yes | **x** | **x** |  |  | **x** |
| No |  |  |  |  |  |
| Can’t tell |  |  | **x** | **x** |  |
| **Apart from the experimental intervention, did each study group receive the same level of care?** | | | | | |
| Yes |  | **x** |  |  | **x** |
| No | **x** |  |  | **x** |  |
| Can’t tell |  |  | **x** |  |  |
| **SECTION C:**  What are the results? | | | | | |
| **Were the effects of intervention reported comprehensively?** | | | | | |
| Yes |  |  |  |  |  |
| No |  | **x** | **x** | **x** |  |
| Can’t tell | **x** |  |  |  | **x** |
| **Was the precision of the estimate of the intervention or treatment effect reported?** | | | | | |
| Yes | **x** | **x** |  | **x** | **x** |
| No |  |  | **x** |  |  |
| Can’t tell |  |  |  |  |  |
| **Do the benefits of the experimental intervention outweigh the harms and costs?** | | | | | |
| Yes |  |  |  | **x** |  |
| No |  |  |  |  |  |
| Can’t tell | **x** | **x** | **x** |  | **x** |
| **SECTION D:**  Will the results help locally? | | | | | |
| **Can the results be applied to your local population/in your context?** | | | | | |
| Yes |  |  |  |  |  |
| No |  |  | **x** |  |  |
| Can’t tell | **x** | **x** |  | **x** | **x** |
| **Would the experimental intervention provide greater value to the people in your care than any of the existing interventions?** | | | | | |
| Yes |  |  |  |  |  |
| No |  |  |  |  |  |
| Can’t tell | **x** | **x** | **x** | **x** | **x** |

No or few limitations: where the assessments for most items in the tool were 'yes'; Minor limitations: where the assessments for most items in the tool were 'yes' or 'cannot tell'; Major limitations: where the assessments for one or more questions in the tool were 'no' (categorization adapted from Pollock et al. [109]).
